# Modeling dependency structures in 450k DNA methylation data

**DOI:** 10.1093/bioinformatics/btab774

**Published:** 2021-11-12

**Authors:** Haakon E Nustad, Ingelin Steinsland, Miina Ollikainen, Emma Cazaly, Jaakko Kaprio, Yuval Benjamini, Kristina Gervin, Robert Lyle

**Affiliations:** Department of Medical Genetics and Norwegian Sequencing Centre, Oslo University Hospital, 0450 Oslo, Norway; Institute of Clinical Medicine, Faculty of Medicine, University of Oslo, 0372 Oslo, Norway; Department of Pharmacy, PharmaTox Strategic Research Initiative, University of Oslo, 0371 Oslo, Norway; Centre for Fertility and Health, Norwegian Institute of Public Health, 0213 Oslo, Norway; Department of Mathematical Sciences, Norwegian University of Science and Technology, 7034 Trondheim, Norway; Institute for Molecular Medicine Finland FIMM, Helsinki Institute of Life Science, University of Helsinki, FI-00014 Helsinki, Finland; Institute for Molecular Medicine Finland FIMM, Helsinki Institute of Life Science, University of Helsinki, FI-00014 Helsinki, Finland; Institute for Molecular Medicine Finland FIMM, Helsinki Institute of Life Science, University of Helsinki, FI-00014 Helsinki, Finland; Department of Statistics and Data Science, The Hebrew University, Mount Scopus, Jerusalem 9190501, Israel; Department of Pharmacy, PharmaTox Strategic Research Initiative, University of Oslo, 0371 Oslo, Norway; Division of Clinical Neuroscience, Department of Research and Innovation, Oslo University Hospital, 0450 Oslo, Norway; Pharmacoepidemiology and Drug Safety Research Group, Department of Pharmacy, Faculty of Mathematics and Natural Sciences, University of Oslo, 0363 Oslo, Norway; Department of Medical Genetics and Norwegian Sequencing Centre, Oslo University Hospital, 0450 Oslo, Norway; Centre for Fertility and Health, Norwegian Institute of Public Health, 0213 Oslo, Norway

## Abstract

**Motivation:**

DNA methylation has been shown to be spatially dependent across chromosomes. Previous studies have focused on the influence of genomic context on the dependency structure, while not considering differences in dependency structure between individuals.

**Results:**

We modeled spatial dependency with a flexible framework to quantify the dependency structure, focusing on inter-individual differences by exploring the association between dependency parameters and technical and biological variables. The model was applied to a subset of the Finnish Twin Cohort study (*N* = 1611 individuals). The estimates of the dependency parameters varied considerably across individuals, but were generally consistent across chromosomes within individuals. The variation in dependency parameters was associated with bisulfite conversion plate, zygosity, sex and age. The age differences presumably reflect accumulated environmental exposures and/or accumulated small methylation differences caused by stochastic mitotic events, establishing recognizable, individual patterns more strongly seen in older individuals.

**Availability and implementation:**

The twin dataset used in the current study are located in the Biobank of the National Institute for Health and Welfare, Finland. All the biobanked data are publicly available for use by qualified researchers following a standardized application procedure (https://thl.fi/en/web/thl-biobank/for-researchers). A R-script for fitting the dependency structure to publicly available DNA methylation data with the software used in this article is provided in supplementary data.

**Supplementary information:**

Supplementary data are available at *Bioinformatics* online.

## 1 Introduction

DNA methylation (DNAm) describes the covalent binding of a methyl group to DNA, which can change the activity of a DNA segment without changing the sequence. DNAm is mitotically heritable, plays a role in the regulation of gene expression (Deaton and Bird, 2011) and is essential for normal development (Li *et al.*, 1992). The most frequent, and most studied, epigenetic mark is 5-methylcytosine, occurring at CpG dinucleotides in humans.

There are numerous methods available for quantifying DNAm levels. The most frequently used are the Illumina Infinium BeadChips comprising the current EPIC array covering approximately 850 000 CpGs and the precursor 450k and 27k arrays (covering 480 000 and 27 000 CpGs, respectively). They provide intensity measures of methylated and unmethylated CpGs, resulting in a ratio defined as M/(M + U + 100). Here, M and U denote the average fluorescent signals from the methylated and unmethylated bead types, respectively. This regularized ratio is usually referred to as the aggregated CpG specific DNAm value.

It is well known that DNAm is spatially dependent along the genome (Affinito *et al.*, 2020; Cokus *et al.*, 2008; Eckhardt *et al.*, 2006; Lister and Ecker, 2009), which is often referred to as co-methylation. There are many ways to define spatial dependency, all of which reflect the similarity between DNAm values at neighboring CpGs. The interest in studying spatial dependency in DNAm is to provide a better understanding of the DNAm machinery, and to leverage the dependence in statistical methods to increase the power and reproducibility of epigenome-wide association studies (EWAS).

Studies of spatial dependency in DNAm are divided into analyses of between-sample and within-sample correlation. Between-sample correlation is the relationship between DNAm at CpG sites across samples, while within-sample correlation refers to the dependency between DNAm at neighboring CpG sites along the genome within one sample. Between-sample correlation studies have focused on identifying regions where DNAm is highly correlated to identify informative CpG sites, which are predictive for DNAm along larger regions (Guo *et al.*, 2017).

Within-sample correlation studies have shown that the strength of the dependence is related to the distance between CpGs. Estimates of the distance where DNAm values are near independent varies from 1 to 3 kb (Lacey *et al.*, 2013; Li *et al.*, 2010; Liu *et al.*, 2014). Most studies have estimated correlation by combining CpG pairs based on the distance between them within a sample and calculating the Pearson or Spearman correlation across those pairs with similar distance measure (Hickey, 2015; Li *et al.*, 2010). Further partition of neighboring CpGs based on genomic context has suggested stronger dependence for CpG sites in CpG islands than outside of islands (Hickey, 2015) and stronger dependence for CpG sites located in gene-associated features than in repeat-associated features (Li *et al.*, 2010).

The approaches described above assume equal population mean at CpG sites at different genomic locations when estimating the correlation within a sample. Here, the population mean is the average DNAm value across samples at a CpG site. The DNAm population mean varies considerably for different CpG sites, with small variation across samples for most sites (Affinito *et al.*, 2020; Xu *et al.*, 2016). Hence, the assumption of equal population mean is violated, and this strongly influences the calculated Pearson or Spearman correlation. A solution to overcome this problem would be to calculate the correlation for each neighboring CpG pair across samples and evaluate how the correlation evolves for increasing distance (Liu *et al.*, 2014), resulting in a combination of between-sample and within-sample correlation. However, in such an approach, differences in dependency structure between individuals cannot be investigated (Hickey, 2015).

Here, we investigated differences in dependency structure for different individuals by focusing on the individual residual processes after removing the observed population mean. This was achieved by subtracting the observed population mean at each CpG site from the individual DNAm value at that site (Xu *et al.*, 2016). By doing this, we could investigate: (i) the dependency structure within samples and (ii) whether differences in dependency structure was associated with technical covariates and biological features such as sex, age and the impact of the genomic sequence.

Our approach is built on modeling spatial dependency as a Gaussian random field with a Matérn covariance function, a common practice within spatial statistics (Cressie, 1993; Diggle *et al.*, 2002). Importantly, this method allows us to include the location of each CpG site in the modeling. In our modeling of the spatial dependency, we obtained estimates of two parameters, κ and τ, for each chromosome describing the dependency structure for each individual. κ influences the strength of the dependence, while τ controls the magnitude of the variation.

## 2 Materials and methods

### 2.1 Samples

The data used in this study came from the Finnish Twin Cohort (FTC), a longitudinal study of three birth cohorts of Finnish Twins (Kaidesoja *et al.*, 2019; Kaprio *et al.*, 2019; Rose *et al.*, 2019). The cohorts consist of 450k DNAm data generated from blood samples from monozygotic (MZ) and dizygotic (DZ) twin pairs (*N* = 1611 samples after quality control). Each twin pair was processed on the same bisulfite conversion plate, and most pairs on the same BeadChip, to minimize technical variation between the co-twins.

The 1611 samples were distributed on 23 conversion plates and 157 BeadChips. Of these samples, 604 unique twin pairs were present; 336 MZ twin pairs and 268 DZ pairs. 254/336 MZ pairs and 230/268 DZ pairs were processed on the same BeadChip. Of the DZ twins, 175 were same-sex pairs, while 93 were opposite-sex pairs. The age distribution of the twins showed 425 individuals above 50 years and 1186 below 40 years, with none in between. For the young population, the median and the interquartile range were 23 and 3, respectively. For the older population, 62 and 7.

### 2.2 Data processing

Initially, sample quality control was performed using MethylAid's (van Iterson *et al.*, 2014) automatic assessment of quality based on five control probe metrics: bisulfite conversion, non-polymorphic sample-dependent control probes, median methylated versus unmethylated signal intensity, sample-independent hybridization control probes and detection *P*-value of negative control probes (*P* > 0.05). The default thresholds were used for each metric and only samples passing all 5 metrics were retained.

Probe quality control was performed by removing ambiguously mapped and poor-quality probes, probes with an intensity value of exactly 0, a detection *P* > 0.01 or bead count < 3 (Zhou *et al.*, 2016). In addition, probes mapped to chromosome X and Y were removed. Only probes with a call rate of 95% or higher across all samples were retained. This resulted in 434 629 probes across the 22 autosomes and 1611 samples with non-missing phenotypic data.

The 450k BeadChip has two different probe designs (type I and II), with different signal distributions. Therefore, when applying a method analyzing regions of data, a frequently used pre-processing step is to map the probe II measurements onto the distribution of probe I measurements. This was done using the BMIQ normalization procedure (Teschendorff *et al.*, 2013). In addition to these preprocessing steps, the beta-values were transformed with the logit2() function to M-values, which are shown to be more homoscedastic and preferred when applying methods to regions of CpGs (Du *et al.*, 2010).

### 2.3 Modeling dependency structure

To investigate differences in dependency structure between individuals, we normalized based on the observed population mean and variance for each CpG. Subtraction of the population mean enabled a focus on the sample-specific residuals for each CpG and an investigation of dependency within samples.

For each individual and each of the 22 autosomes, the following model was used:

$$\begin{array}{c} y|\mu ,\sigma_{0}^{2}∼\prod_{p=p_{1}}^{p_{n}}N(y_{p};\mu_{p},\sigma_{0}^{2})\;\;\;\;\;\;\;\;\;\;\;\;\;\;\;\;\;\;\;(\text{Likelihood})\\ \mu =\beta +\xi \\ µ|\tau ,k∼N(\beta ,Q^{-1}(\tau ,k))\;\;\;\;\;\;(\text{Latent}\;\text{Gaussian}\;\text{field})\\ \sigma_{0},\tau ,k∼\pi (\sigma_{0},\tau ,k)\;\;\;\;\;\;\;\;\;\;\;\;\;\;\;\;\;\;\;\;\;\;\;\;(\text{Hyperparameters}) \end{array}$$

This yielded 22 independent parameter estimates for each sample. As previously described, y_p_ is the discrepancy from the population mean for a sample at CpG site with base pair position p. β is a sample-chromosome-specific intercept and **ξ** is a random effect fitted to the residuals following a dependency structure along the chromosomes where τ and κ are the sample-chromosome-specific dependency quantification parameters. σ_0_, τ and κ are the hyperparameters in our model, where the exact prior specifications are given in Section 2.4.

The random effect **ξ** was modeled as a Gaussian random field with mean zero and covariance matrix Q^−1^ defined by a Matérn covariance function, given by

$$\mathrm{Cov}\left(\xi \left(p_{1}\right),\xi \left(p_{2}\right)\right)=\frac{\sigma^{2}}{\Gamma \left(\lambda \right)2^{\lambda -1}}{\left(\kappa \left|p_{1}-p_{2}\right|\right)}^{\lambda }\cdot K_{\lambda }\left(\kappa \left|p_{1}-p_{2}\right|\right),$$

where σ^2^ is the marginal variance in the spatial white noise process and |p_1—_p_2_| is the absolute distance in base pair between location p_1_ and p_2_.

K_λ_(κ|p_1_ – p_2_|) denotes the modified Bessel function of the second kind and order λ. With λ, we choose what type of Matérn covariance function we assume; for this study, we set λ = 0.5 which specifies exponential decay. This is based on correlation function estimations from Li *et al.* (2010) and Hickey (2015), which seemed to follow an exponential shape. With λ treated as fixed, κ and τ are the main parameters of the inference that describe the dependency structure.

The scale parameter κ is most easily interpreted through the range parameter r, where

$$r=\frac{\sqrt{8\lambda }}{\kappa }.$$

The range r is the distance at which the spatial correlation is close to 0.1 (Blangiardo and Cameletti, 2015, p. 194). The marginal variance σ^2^ and the variance controlling parameter τ have an inverse relationship, shown through the following formula:

$$\sigma^{2}=\frac{\Gamma \left(\lambda \right)}{\Gamma \left(\lambda +0.5\right)\sqrt{4\pi }\kappa^{2\lambda }\tau^{2}}.$$

Here, Γ(·) is the gamma function. Of note, the marginal variance is dependent on both κ and τ.

To do computationally efficient Bayesian inference, the integrated nested Laplace approximations (INLA) R package (www.r-inla.org) (Rue *et al.*, 2009) was used. The Stochastic Partial Differential Equation (SPDE) (Lindgren *et al.*, 2011) was used for the spatial modeling. A detailed description of the approach is found in the studies by Lindgren *et al.* (2011) and Blangiardo and Cameletti (2015, pp. 194–197). INLA and SPDE include implementation for 1-dimensional Gaussian random fields as the current study, although most literature and presentations of this software focus on 2-dimensional and 3-dimensional Gaussian random fields.

### 2.4 Prior specifications

For β and σ_0_, we used the default priors given by INLA, β ∼ *N*(0, 10^6^) and $1/\sigma_{0}^{2}\;$ ∼ Gamma(1, 0.00005). In agreement with the parametrization in INLA, we specified the priors for τ and κ on a log scale. This resulted in the following priors:

$$\text{log}\;\left(\tau \right)∼N\left(\text{log}\;\left(\tau_{m}\right),0.05\right)$$

 

$$\text{log}\;\left(\kappa \right)∼N\left(\text{log}\;\left(\kappa_{m}\right),0.05\right)$$

τ_m_ and κ_m_ are defined using range parameter equal to 3000 (Lacey *et al.*, 2013) and σ^2^ = 1 in the spatial white noise process. This resulted in the following mean estimates for the priors; τ_m_ = 27.39 and κ_m_ = 0.00067. The precision parameter 0.05 is equivalent to a large variance, ensuring a wide prior distribution.

### 2.5 Intra-class correlation coefficients

Intra-class correlation coefficients (McGraw and Wong, 1996) (ICCs) were used to investigate the amount of total variation in the posterior mean estimates of the dependency parameters that could be explained by variation between individuals. These were calculated by seeing each individual’s posterior mean estimate from the different chromosomes as replicates. The *icc()* function from package irr with specifications *model = oneway*, *type = absolute agreement* and *unit = single* was used to calculate the ICCs.

### 2.6 Deviance information criterion

To assess the goodness of fit, the deviance information criterion (Spiegelhalter *et al.*, 2002) (DIC) was used to compare the plain model to the dependency model for each fitting, i.e. for each individual and chromosome. The DIC is a trade-off between the goodness of fit and the model complexity. A lower value indicates a better fit to the data. A model with a difference greater than 4 to the best model is to be viewed as having considerably less support (Spiegelhalter *et al.*, 2002).

## 3 Results and discussion

In total, we obtained results from 35442 models (1611 individuals × 22 chromosomes). By comparing the DIC between the plain and dependency model, the dependency model was favored for all individuals and chromosomes (Supplementary Fig. S1).

For each parameter of interest, β (sample-chromosome-specific intercept), τ and κ or r and σ (sample-chromosome-specific dependency quantification parameters), we obtained marginal posterior distributions. The spatial dependency is described by either τ and κ, or r and σ. The reason for using both sets of parameters is that r and σ are easier to interpret, while log(τ) and log(κ) are better for statistical testing due to close to normal distributions. τ and κ are handled at a log-scale to increase interpretability, since the modeling mesh (the base pair location of each CpG) makes τ large and κ small. For the variance controlling parameter σ, a larger value would indicate more variation among the residuals **ξ**. In terms of τ, which is a precision parameter, a smaller value would indicate more variation. For the parameters r and κ, a larger r and a smaller κ, results in smoother residuals **ξ**, that is stronger dependence.

### 3.1 Differences in dependency structure across individuals

**Fig. 1.**
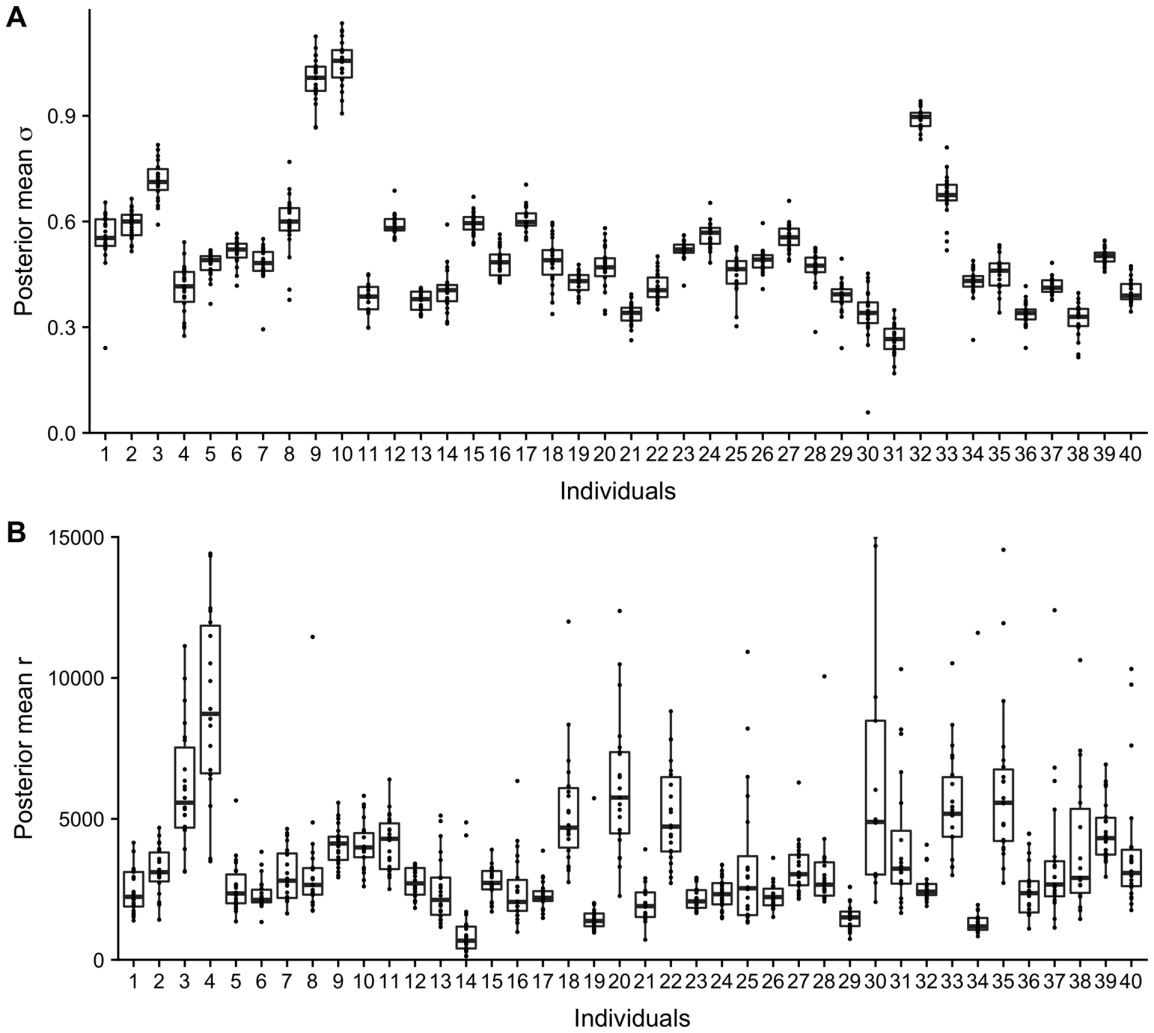
Boxplots of posterior mean estimates of marginal standard deviation σ (**A**) and range *r* (**B**) from the dependency modeling for a subset of 40 randomly chosen individuals. Each individual-specific box is calculated based on estimates from the 22 autosomal chromosomes. The size of each box reflects the variability within the individual, whereas the variability between boxes reflects differences between individuals

The dependency structure parameters revealed a consistent pattern across chromosomes within individuals. This pattern was most evident in the posterior mean of σ, but was also observed in the posterior mean of the range r for most individuals (Fig.  1A and B). The dependency structure differed between individuals, which suggest there are underlying biological and/or technical features influencing the dependency parameters. By including the uncertainty of the parameters, the differences between the individuals are still evident (Supplementary Fig. S2A and B). Together, this indicates genome-wide stability of the dependency structure within individuals, and differences between individuals. The estimated correlation functions using the posterior mean estimates from chromosome 19 shows how the dependence is stronger for larger r (smaller κ, Supplementary Fig. S2C).

In addition to visual inspection, we estimated the amount of total variation in the posterior mean estimates that could be explained by variation between individuals by calculating ICCs. This was done separately for log(τ) and log(κ), and using one randomly chosen individual from each unique family (748 individuals). The resulting ICC confidence intervals were estimated to be ICC_log(τ)_ = [0.651, 0.697] and ICC_log(κ)_ = [0.360, 0.412], indicating that a substantial part of the variation was attributed to variation between individuals for both parameters. The ICC specification we used assumes each individual posterior mean for the different chromosomes to be replicates. Although this is a simplification, we do observe that their estimates are similar. Furthermore, this assumption is conservative in that it produces an ICC estimate that undershoots the true value.

### 3.2 Differences in range across chromosomes

**Fig. 2.**
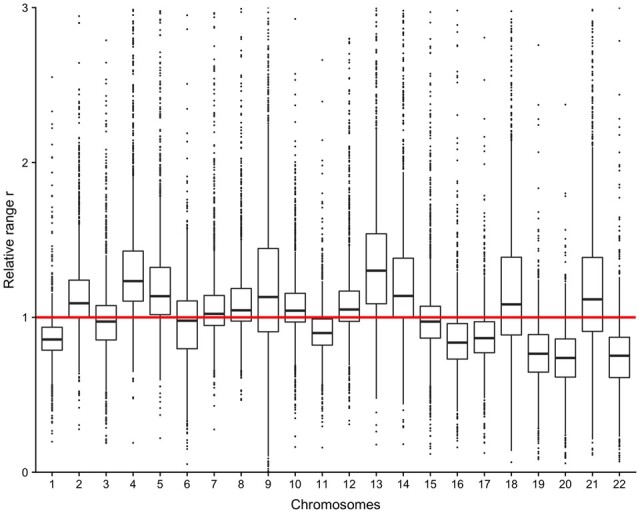
Boxplot of relative range for the 22 autosomal chromosomes. For each individual, each posterior mean estimate of the range is divided by the median of the estimates. 999 of ∼27 000 points omitted, because they are outside the domain [0,3]. 1 is marked with a red line

Since chromosomes differ in features such as length and gene density, we investigated whether the range (r) estimate showed chromosomal differences. Since r showed differences across individuals, we normalized each individual’s r estimates by dividing with its median r estimate across chromosomes. This enabled comparison of r estimates per chromosome across individuals. The results revealed differences between the chromosomes, indicating that the dependency varies in terms of strength (Fig.  2). The same relationship was not observed for the posterior mean of σ.

The Spearman correlation between the median relative r parameter from chromosome 1–22 and the relative gene density was equal to –0.81 (95% confidence interval: [-0.92, –0.60]), indicating a highly significant inverse relationship (*P* = 3.9e-06). The relative gene density was calculated by dividing the gene density for each chromosome (Mayer *et al.*, 2005) with the median gene density. This suggests that lower gene density results in a higher r estimate, indicating a stronger dependence between adjacent CpGs. This might be due to less gene-rich chromosomes being on average more densely packed, with higher ratio of heterochromatin to euchromatin (Gilbert *et al.*, 2004). This can influence the spatial distance between CpG sites and thus influence the dependency structure.

Another possible explanation could be the proportion of CpG sites found in islands compared with outside islands on the different chromosomes. This was investigated by calculating the spearman correlation between the relative (compared with the median proportion) proportions of CpG sites found in islands for the different chromosomes from the hg19 annotation file and the median relative r parameter for chromosome 1–22. The resulting estimate was equal to -0.69 (95% confidence interval: [–0.86, –0.38]), indicating a significant, inverse relationship (*P* = 5e-04). As many genes are related to promotor regions with several CpG islands, similar relationships with r were expected. Previous studies of the dependency structure without removing the population mean found an opposite relationship; the correlation function for CpGs in islands had a larger range parameter than the correlation function for CpGs outside of islands (Hickey, 2015; Liu *et al.*, 2014). However, their estimation of the dependency structure is highly influenced by the population mean. Therefore, the results are not directly comparable. This warrants further research with a more advanced model, distinguishing the dependency within a CpG island and the dependency outside of islands by introducing an island effect to both τ and κ. Such a model can be fitted to both the population mean and the sample-specific residuals, to analyze the differential effect of the islands and to compare with previous studies. We leave this to future work.

### 3.3 Technical variation in spatial dependency: differences across conversion plates

**Fig. 3.**
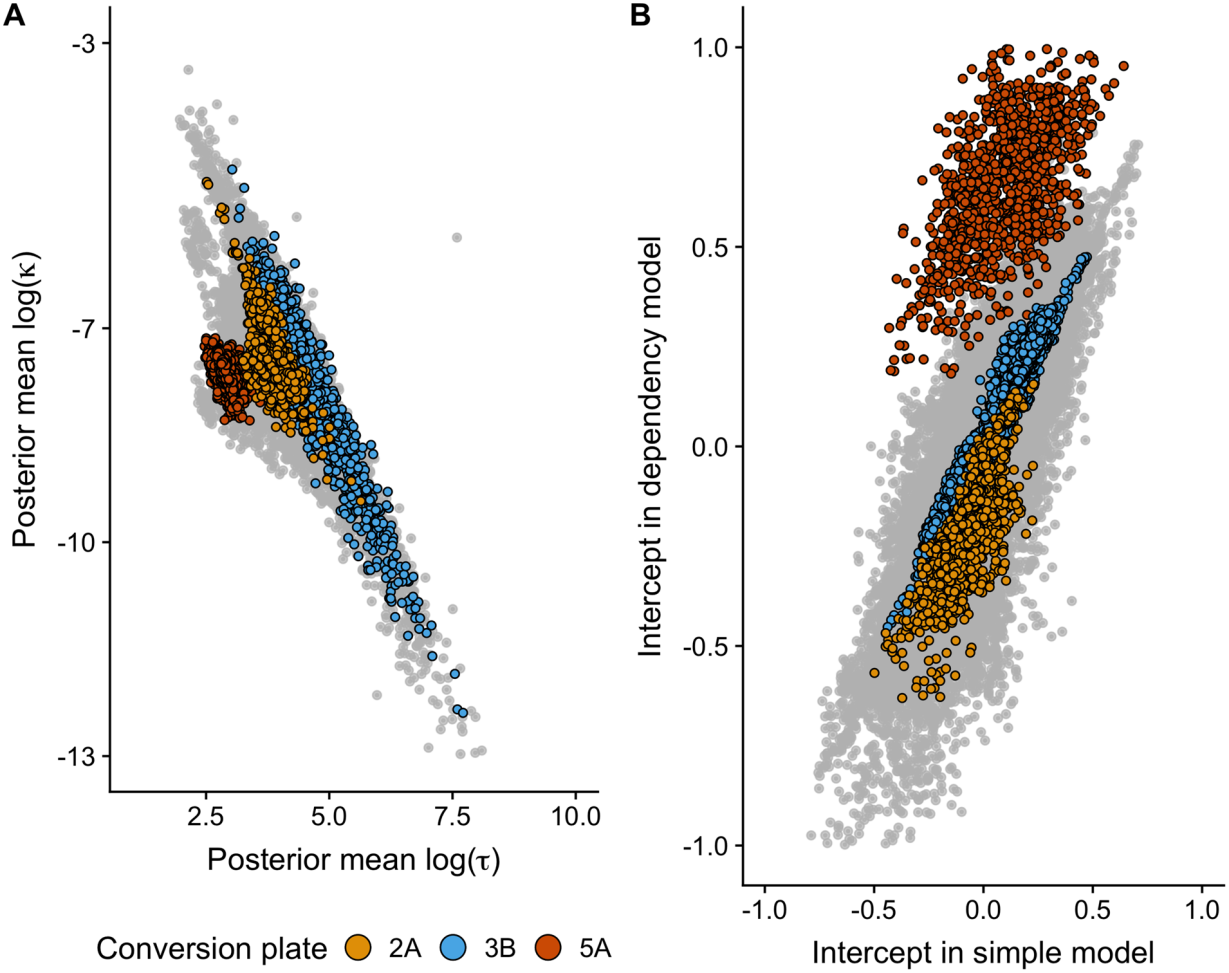
(**A**) Posterior mean of log(κ) plotted against log(τ) for each individual and chromosome. Three conversion plates have been highlighted. (**B**) Intercept in the dependency model plotted against the intercept in the plain model for each individual and chromosome. The same three conversion plates have been highlighted

It is known that DNAm measurements are subject to batch effects. This can be due to differences in laboratory procedures, particularly bisulfite conversion rates (Assenov *et al.*, 2014). Differences based on conversion plates can be seen in the dependency parameters (Fig.  3A) and in the distribution of the intercepts from each model showing mean and variance differences (Fig.  3B). The difference in intercepts based on conversion plate is clearest in the dependency model. This effect was especially evident for plate 5A, which had a clear mean shift in log(τ), log(κ) and intercept. Of note, the dependency parameters seem to have a linear relationship, especially for the more outlying samples (Fig.  3A).

We further investigated conversion plate and BeadChip effects by calculating the squared difference between the dependency parameters for different pairings of the individuals:

$$D_{\tau ,j}=\left({\left(\text{log}\;\tau_{\mathrm{Twin}\;1,j}-\text{log}\;\tau_{\mathrm{Twin}\;2,j}\right)}^{2}\right)\forall \mathrm{Twin}\;\mathrm{pairs},\;\mathrm{chr}\;j,$$

 

$$D_{\kappa ,j}=\left({\left(\text{log}\;\kappa_{\mathrm{Twin}\;1,j}-\text{log}\;\kappa_{\mathrm{Twin}\;2,j}\right)}^{2}\right)\forall \mathrm{Twin}\;\mathrm{pairs},\;\mathrm{chr}\;j.$$

Here, D_*,j_ is the difference in log(*) between the pairing of individuals calculated for each chromosome (chr) 1-22. The pairings considered were: (1) pairing of Twin 1 and Twin 2, (2) random pairing of individuals, (3) random pairing matched on conversion plate and (4) random pairing matched on conversion plate and BeadChip (Supplementary Fig. S3A and B). In Supplementary Table S1, the results of a Mann–Whitney test comparing 1–4 are given for each chromosome. In addition, a combined *P*-value was calculated using an extended Fisher’s method for correlated tests (Dai *et al.*, 2014), since chromosomal estimates from the different individuals showed significant ICCs. These combined *P*-values are given in Table  1.

**Table 1.** Mann–Whitney one-sided test results

Mann–Whitney test, alternative hypothesis	Number of pairs	Combined *P*-value	Combined *P*-value
		(log(tau))	(log(kappa))
Random pairing of individuals greater differences than random pairing matched on conversion plate	N_RandomPairs_ = 604	1.14E-10	5.77E-03
	N_MatchedPlate_ = 604		
Random pairing of individuals matched on conversion plate greater differences than random pairing matched on conversion plate and BeadChip	N_MatchedPlate_ = 604	3.82E-04	2.17E-02
	N_MatchedPlateBeadChip_ = 604		
Random pairing of individuals matched on conversion plate greater differences than true pairing of twins	N_MatchedPlate_ = 604	2.26E-10	2.54E-10
	N_TruePairing_ = 604		
Random pairing of individuals matched on conversion plate and BeadChip greater differences than true pairing of twins	N_MatchedPlateBeadChip_ = 604	2.04E-04	3.40E-06
	N_TruePairing_ = 604		
Monozygotic twins run on different BeadChip greater differences than monozygotic twins run on same BeadChip	N_DifferentBeadChip_ = 82	2.15E-01	9.22E-01
	N_SameBeadChip_ = 254		
Dizygotic twins run on different BeadChip greater differences than dizygotic twins run on same BeadChip	N_DifferentBeadChip_ = 38	8.65E-02	8.96E-01
	N_SameBeadChip_ = 230		
Dizygotic twins greater differences than monozygotic twins	N_Dizygotic_ = 268	2.92E-03	9.76E-06
	N_Monozygotic_ = 336		
Opposite sex dizygotic twins greater differences than same sex dizygotic twins	N_OppSex_ = 93	3.17E-02	1.64E-02
	N_SameSex_ = 175		

*Note*: These test results are combined across chromosomes with the extended Fisher’s method for correlated tests. N_*_ is the number of twin pairs in the respective groups. E–X is used for 10^-X^.

The following order from smallest differences to largest was found for the pairings: twin pairs (1), individuals matched on conversion plate and BeadChip (4), individuals matched on conversion plate (3), random pairing of individuals (2). From the relevant comparisons given in Table  1, conversion plate is more strongly associated with variation in dependency parameters than BeadChip. This is seen by comparison 3–2 yielding very significant decrease in differences, while 3–4 not yielding large significance. In addition, MZ and DZ twins run on different BeadChips were compared against MZ and DZ twins run on same BeadChips, to further investigate BeadChip differences. Although the sample size is limited, the combined *P*-value does not suggest larger differences in dependency parameters for twins run on different BeadChips. Since comparison 3–1, and 4–1, showed significant differences, biological/genetic differences are likely to influence the dependency parameters. The random pairings were done 100 times, and the median distribution of these, ranked by their median, were used for figures and tests.

Differences in dependency structure parameters associated with conversion plate suggested not only intercept changes across chromosomes and genomes, but also patterns of small DNAm differences throughout chromosomes which in combination could result in the observed differences in dependency parameters. We observed differences in both log(τ) and log(κ) associated with conversion plate, indicating differences in both the amount of variation in the spatial process and the strength of the dependency. Conversion plate 5A showed most distinct distribution of dependency parameters. Interestingly, this is the only plate processed at a different core facility.

### 3.4 Biological variation in spatial dependency

In addition to technical variation in spatial dependency, we investigated biological variation by studying the impact of genetics, sex differences and differences between age groups.

#### 3.4.1 Impact of genotype

MZ twins are genetically identical at the sequence level, while DZ twins share on average 50% of their segregating genes. To investigate the impact of genotype, the squared differences between the parameters were calculated for MZ and DZ twin pairs separately. The MZ twins showed smaller differences between the parameters than the DZ twins (Supplementary Fig. S4A and B). The difference between the distributions was tested with a Mann–Whitney test, which resulted in a significant location (mean) shift (Table  1).

Studies of methylation quantitative trait loci (Kerkel *et al.*, 2008; Zhang *et al.*, 2010) have shown that genotype influences DNAm at many CpG sites, making the DNAm profiles more similar between MZ twins than DZ twins. Therefore, more similar dependency parameters were expected between MZ twins compared to DZ twins. However, as 1/3 of the DZ twins were opposite-sex pairs, sex-differences could explain some of the differences shown for genotype.

#### 3.4.2 Sex

In addition, we analyzed DZ twins with same sex versus opposite sex, and investigated the distributions of squared differences (Supplementary Fig. S4C and D). Opposite-sex twin pairs had larger differences in both dependency parameters, shown with a weak, significant association in Table  1 (*P*-value between 0.01 and 0.05). Since opposite-sex twin pairs showed larger within-pair differences than same-sex twin pairs, some of the variation seen in the dependency parameters could be explained by sex differences. Several CpG sites and regions genome-wide have been shown to be associated with sex (Liu *et al.*, 2010; Yousefi *et al.*, 2015), which in combination could result in the differences in dependency structure on a chromosomal level.

#### 3.4.3 Age

**Fig. 4.**
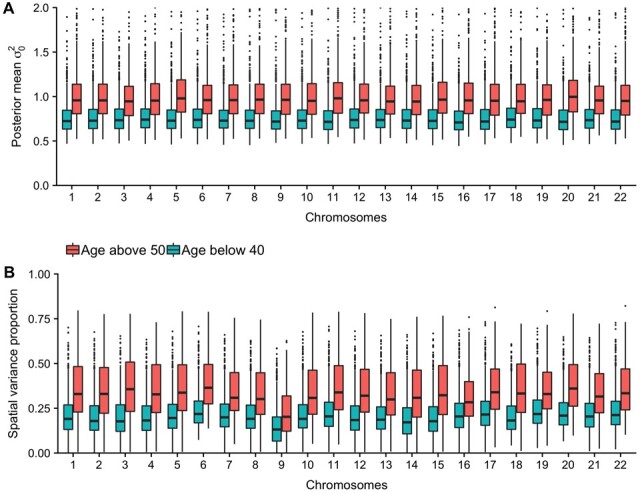
(**A**) Posterior mean of the residual variance in the plain model, colored on age groups. (**B**) Proportion of variance explained with a spatial effect in the model, colored on different age groups. Calculated by taking 1 minus the residual variance in the spatial model divided by the residual variance in the plain model

Since the individuals are from two age groups, we investigated the distribution of log(τ) and log(κ) for each chromosome for the individuals below 40 years against the individuals above 50 years (Supplementary Fig. S5). For the variance controlling parameter log(τ), a clear shift is seen between the two groupings for all chromosomes. This shift is toward lower value for the older population, indicating more variation absorbed by the spatial dependency effect. This could be caused by older individuals having larger variation across their epigenome, influencing the amount of possible variation that can be absorbed by the spatial effect (Fig.  4A). However, when investigating the proportion of variance explained by the spatial process compared with the total variance for each individual, we still observed a larger proportion for the older individuals for all chromosomes (Fig.  4B).

Every exposure affecting the epigenome influences DNAm across sites and regions. As the underlying dependence seem to be different for different individuals (Fig.  1A and B), each individuaĺs DNAm pattern might be influenced differently. Throughout years of different exposures, these accumulated small differences in DNAm can strengthen the underlying dependency pattern and increase the possibility of distinguishing the dependency process from the random noise. This might explain why the proportion of variation accounted for in the spatial process is larger for older individuals (Fig.  4B). It may also be due to stochastic mitotic events that yield small cumulative differences over time, establishing unique correlated patterns for each individual.

## 4 Conclusion

In this study, we used a flexible modeling framework to quantify the within-sample dependency structure of DNAm in autosomal chromosomes. For all individuals and chromosomes, the dependency model was favored by the DIC supporting existence of within-sample dependency in DNAm. The dependency parameters were consistent across chromosomes within individuals, and showed differences between individuals. The differences between individuals were most strongly associated with bisulfite conversion plate. Hence, bisulfite conversion plate should be considered when correcting for or leveraging dependency structure in array-based DNAm studies. Interestingly, individual differences in spatial dependency were associated with age, genotype and sex across autosomal chromosomes. The proportion of variation accounted for by the spatial process was larger for older individuals, indicating that accumulated environmental exposures and/or stochastic events may have a unique influence on DNAm for each individual. This results in small individual differences in CpG methylation, giving rise to patterns that are recognizable over large regions such as chromosomes.

## Funding

This work was supported by the Research Council of Norway [241117, 250362] (H.E.N., K.G., R.L. and I.S.); the Academy of Finland [308248, 312073, 307339, 251316]; the Sigrid Juselius Foundation (M.O., J.K. and E.C.); and National Institutes of Health [R01GM083084] (Y.B.).

*Conflict of Interest*: none declared. 

## Supplementary Material

btab774_supplementary_data
