# Supplementary figures and images for: Modeling dependency structures in 450k DNA methylation data
Source: Bioinformatics. 2021 Nov 12;38(4):885–91. doi: 10.1093/bioinformatics/btab774 (PMC8796368; doi:10.1093/bioinformatics/btab774)

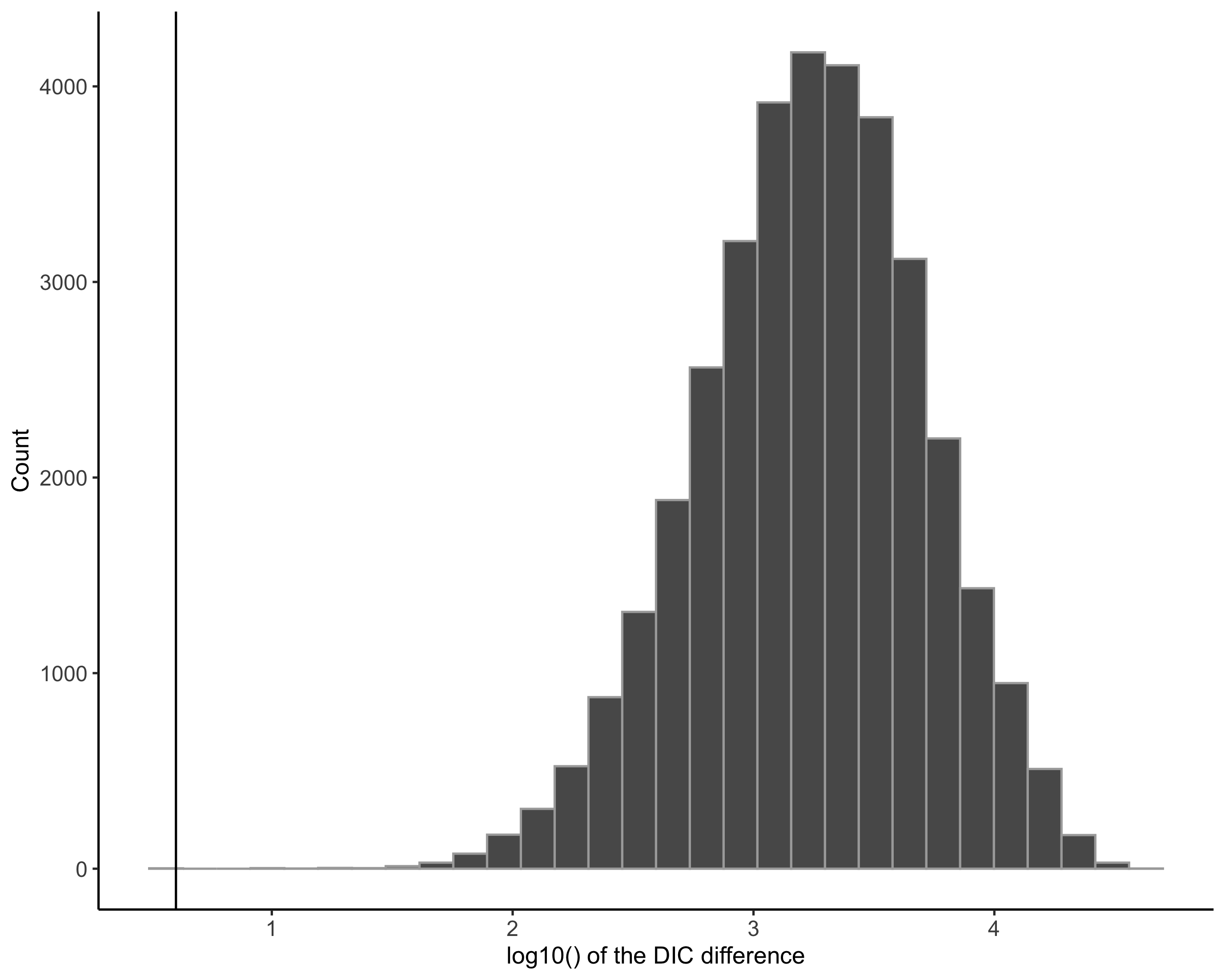

Supplement: btab774_supplementary_data [file btab774_supplementary_data.zip › OP-CBIO210786_PECorr_AttachmentsFolder_Supplementary_Figure_1[AU].tiff]

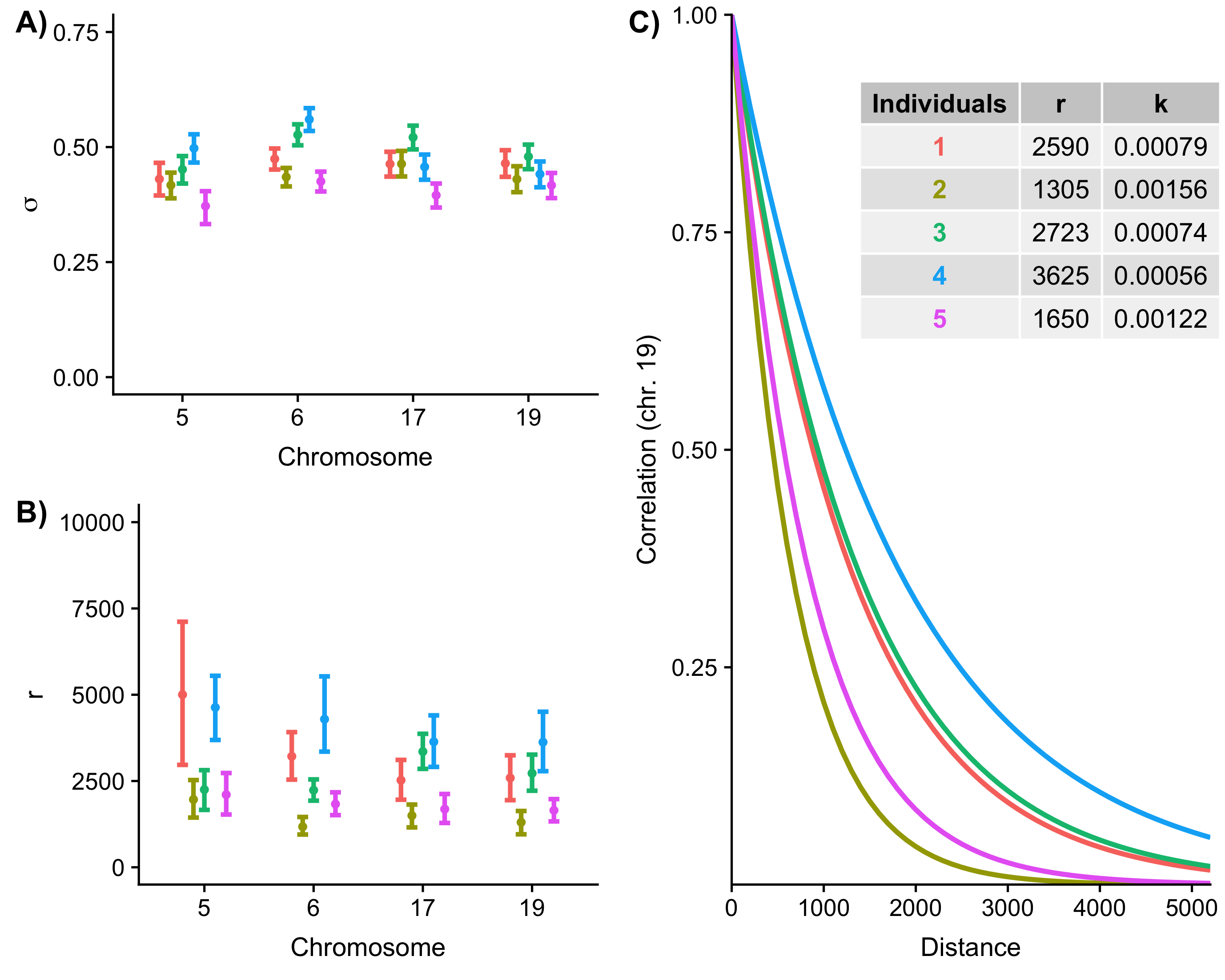

Supplement: btab774_supplementary_data [file btab774_supplementary_data.zip › OP-CBIO210786_PECorr_AttachmentsFolder_Supplementary_Figure_2[AU].tiff]

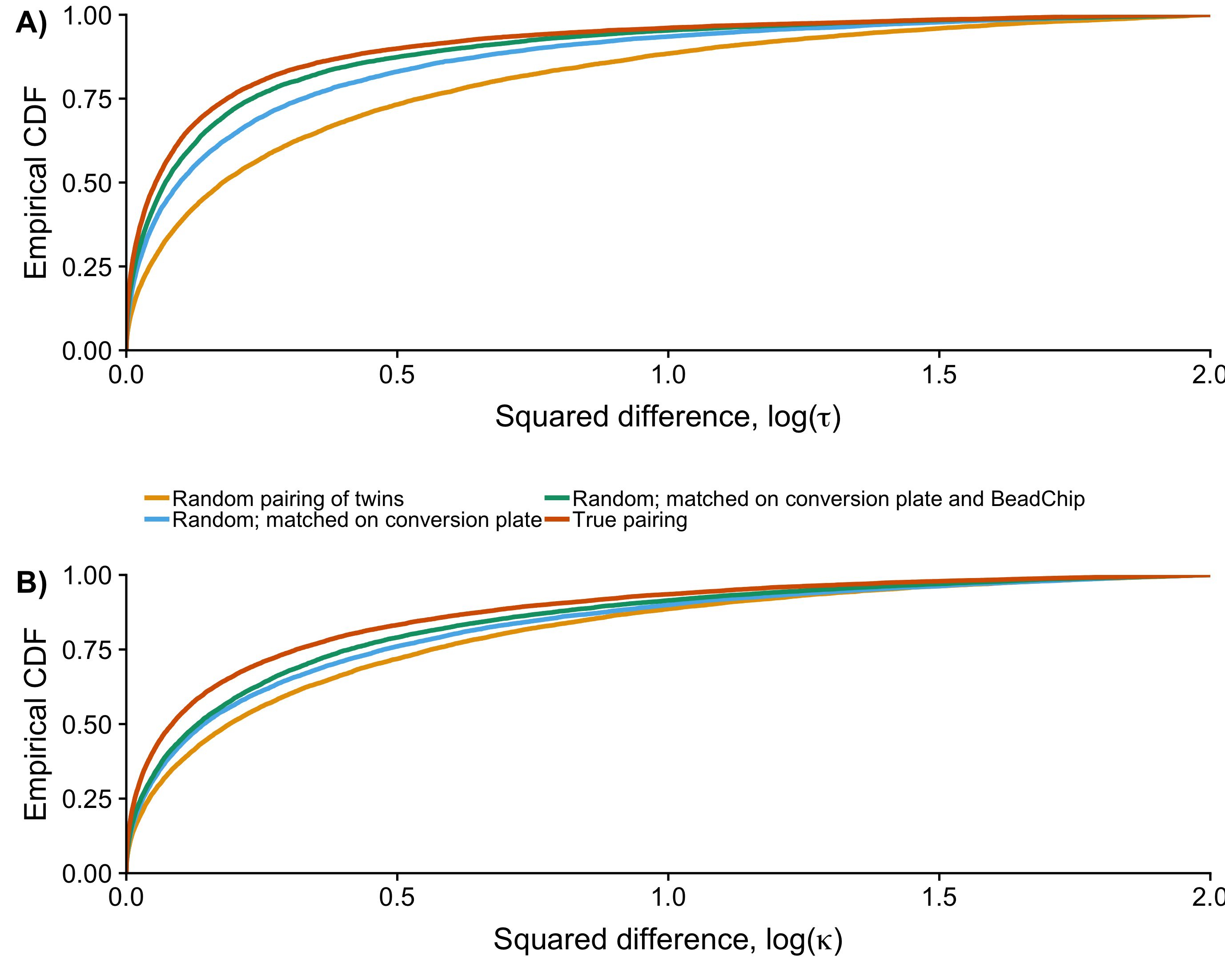

Supplement: btab774_supplementary_data [file btab774_supplementary_data.zip › OP-CBIO210786_PECorr_AttachmentsFolder_Supplementary_Figure_3[AU].tiff]

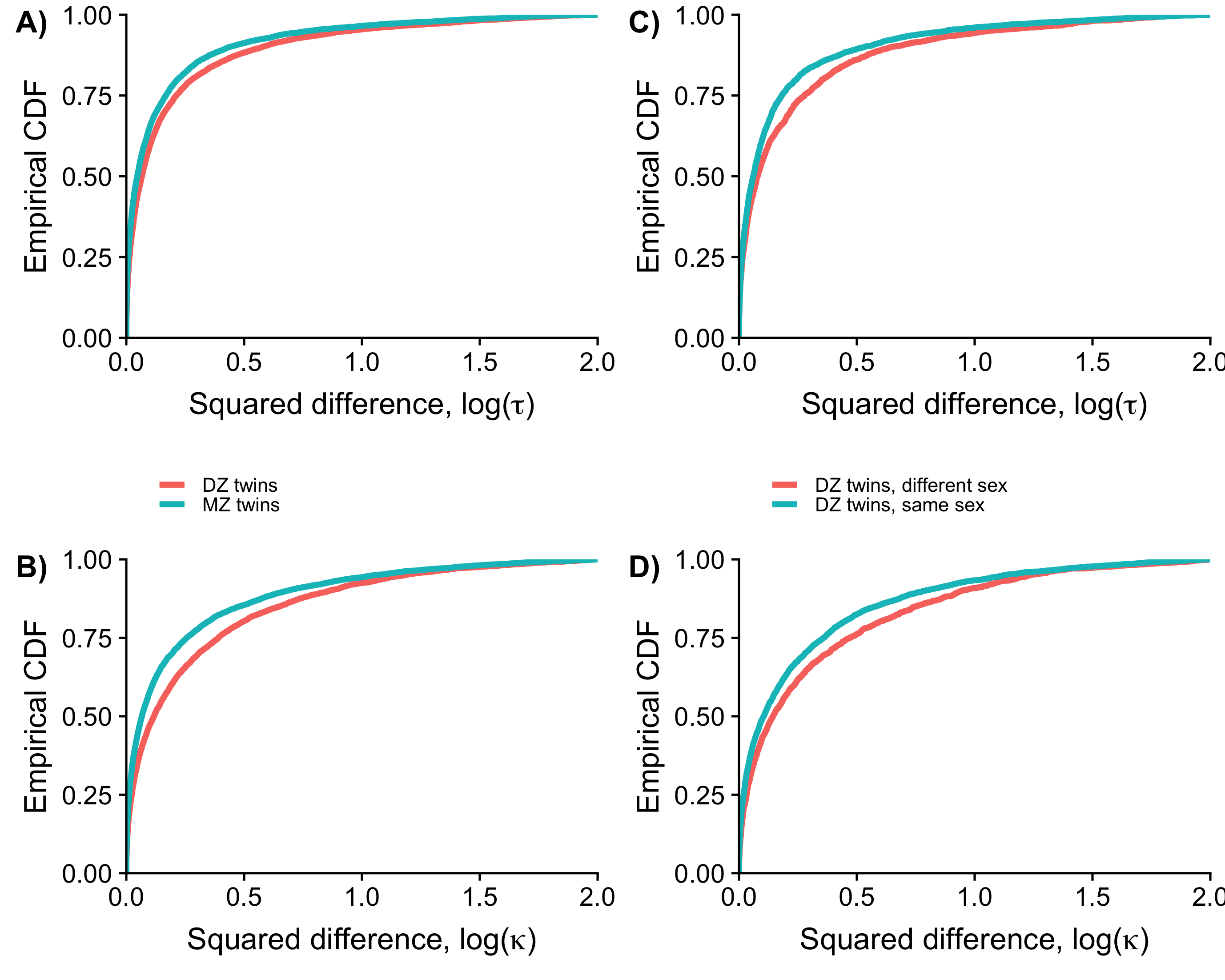

Supplement: btab774_supplementary_data [file btab774_supplementary_data.zip › OP-CBIO210786_PECorr_AttachmentsFolder_Supplementary_Figure_4[AU].tiff]

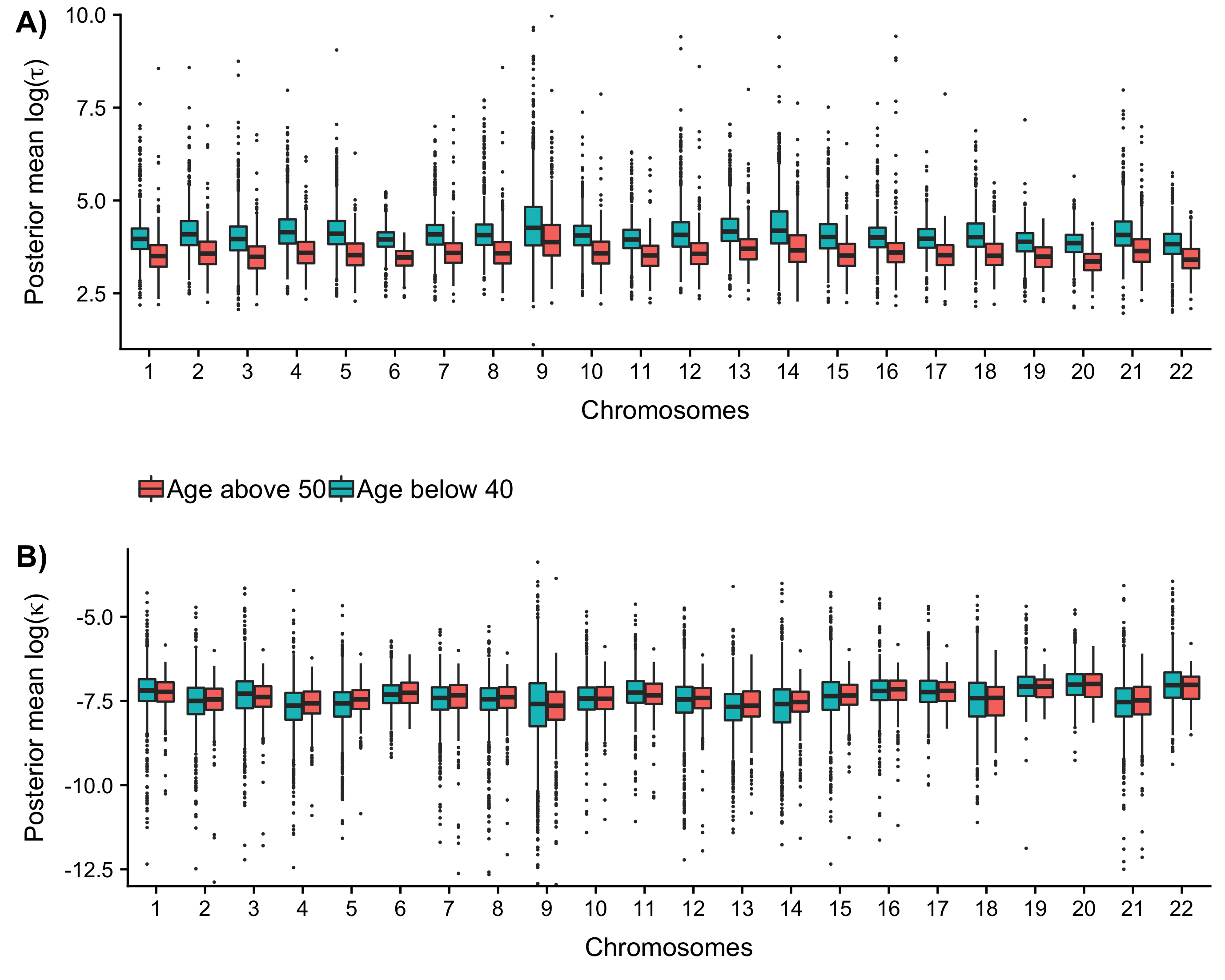

Supplement: btab774_supplementary_data [file btab774_supplementary_data.zip › OP-CBIO210786_PECorr_AttachmentsFolder_Supplementary_Figure_5[AU].tiff]
